# The Spirit of Co-Operation

**Published:** 1918-12-07

**Authors:** 


### THE MATRONS' AND SISTERS' DEPARTMENT.

### THE SPIRIT OF CO-OPERATION.

### Working for Service, not for Self.

So much is being written and said about the training of nurses and the merits and defects of different systems, that it is not always easy to keep thought clear as to the end in view, and it may be well to pause for a moment and give ourselves time to think back to first principles.

After all, when closely examined, of what does training consist? Is it of learning to give certain treatment, to prepare patients for operation, to dress wounds, and so forth? If this were all, human machines might indeed be quite satisfactory, and the god of efficiency be a suitable deity to be worshipped in the training-school. But are not these things in reality only part of a far larger whole? Grouped loosely, a nurse should be trained in reference to, first, the patient's surroundings; secondly, his personal comfort; thirdly, his atmosphere; and fourthly, the medical and surgical treatment ordered for him. We hear much discussion nowadays with regard to what is called "menial" work, and many protests against its enforcement upon nurses. Quite apart from the fact that no work that is right can possibly be menial or degrading—"Who sweeps a room as for God's laws, Makes that and the action fine"—there is, back of all, a very real and adequate reason for all this sweeping and dusting and washing and polishing which many seem to find so irksome. For one of the main requisites as regards the patient's surroundings is cleanliness, and although perhaps 30 or 40 per cent. of the probationers to be trained may fully understand the importance of such cleanliness, and require no training to fit them to see it carried out, the other 60 or 70 per cent. will not understand it, and would be incapable of enforcing it unless they had been through every step themselves and had been compelled to realise its importance by being made to do it thoroughly and regularly. But if the nurse is given no reason for it all, then she does become simply a machine, accomplishing so many revolutions per diem. On the other hand, make your probationer realise at the outset that she is working directly for the patient by keeping his surroundings fresh and wholesome, that she is not washing the window-ledges or putting the castors straight just because Matron or Sister may come along and see the one dirty or the other turned the wrong way, and find fault with her, but in order to help the patient to get well by making it impossible for harmful microbes to dwell near him, and by eliminating the possibility of someone stumbling over the castor and giving him a jar; then you will have gone far towards changing her whole attitude to her work.

The spirit of co-operation will accomplish marvels. The writer used often to wonder why one particular ward in her hospital was always so happy. It was not that it was easy—on the contrary, it was one of the heaviest; nor was it only that Sister was an excellent teacher, and that she worked harder than any of her nurses, nor that she was kind and considerate to them. The reason generally given by the nurses was that everyone worked, and that things were done so methodically. The writer has since realised that it was because everyone worked *with* Sister *for* the patients that no one ever heard a grumble, and that more work was accomplished than in any other ward, while the smallest thing well done—even to the cleaning of the inkstands—was noticed, and a timely word of praise given which made the recipient put more elbow-grease than ever into her polishing the next day: on the other hand, the smallest thing badly done was likewise noticed, and corrected. At the same time, each nurse was individually taught and trained in the various processes of nursing that went on in the ward, so that she felt that she had a very real part in the economy of the whole.

If this spirit of co-operation were to be more actively taught and practised in hospital wards, work at present characterised as drudgery would be seen in its true proportion, and cease to be the terrible burden it now appears, while at the same time boundless opportunities of training would be opened up. For the co-ordination of every part of hospital work could be pointed out, and the nurse could be taught to see that each stage of her training has a direct bearing upon one or other of the groups above-mentioned—whether it be sweeping or dusting, bed-making, feeding, arranging pillows, filling hot bottles, being gentle, quiet, and cheerful, or giving drinks and medicines, dressing wounds, or carrying out medical treatment.

At the same time, the loftiness of spirit which should characterise the profession would be preserved by keeping more clearly in view the aim and object of nursing, which, in a word, is the restoration to health of the patient. Once set this in the forefront of all teaching and training, and it will become apparent that nothing in hospital is an isolated task imposed from without, but that matron, sisters, nurses, doctors are all working together for service, not for self.

